# In Memoriam BJPsych Bulletin In Memoriam List 2022

**DOI:** 10.1192/bjb.2021.117

**Published:** 2022-02

Akpuaka, Francis Chinedu

Affiliate

Arie, Thomas Harry David Honorary

Fellow

Backer, Hana

Affiliate – Over 40 Years member

Bangash, Saifullah Khan

Member

Baruch, Ury Bernard Helmut Foundation

Member

Bearcroft, Rosalind Irene

Fellow – Over 40 Years member

Beer, Elaine Catherine

Fellow – Over 40 Years member

Bergmann, Klaus

Fellow – Over 40 Years member

Binneman, Brendon Michael

Member

Briscoe, Oliver Villiers

Fellow – Over 40 Years member

Brown, Philip Morrison

Fellow

Caldicott, Fiona

Fellow

Chan, Chee Hung

Member

Choudhury, Abdul Fowad

Affiliate

Choudry, Nas

Fellow – Over 40 Years member

Coia, Denise Assunda Honorary

Fellow

Davenport, Susan Lesley

Member – Over 40 Years member

Davies, Brian Michael

Fellow – Over 40 Years member

De-Groot, Michael Henry Foundation

Member

Dennehy, Constance Marguerita

Fellow – Over 40 Years member

Devakumar, Vijayapura Chicknanjanna

Member – Over 40 Years member

de Wet, Cornelis

Member

D'Orban, Paul T

Fellow – Over 40 Years member

Duddle, Constance May

Fellow – Over 40 Years member

Dunlop, Joyce Lilian

Fellow – Over 40 Years member

Edwards, Wayne John

Member

Fielding, John Mathew

Member – Over 40 Years member

Fine, Stuart Hamilton

Fellow – Over 40 Years member

Freed, Edgar David

Fellow – Over 40 Years member

Gaitonde, Alison Myra

Member – Over 40 Years member

Gath, Ann Mary Gethin

Fellow – Over 40 Years member

Hewland, Helen Robyn

Fellow – Over 40 Years member

Hickling, Frederick W

Fellow – Over 40 Years member

Hilary-Jones, Evan Peter

Fellow

Hill, Oscar William

Fellow – Over 40 Years member

Hore, Brian

Fellow – Over 40 Years member

Horrocks, Frederick Arthur

Member

Hutchinson, Doreen

Member – Over 40 Years member

Hughes, John Samuel

Fellow – Over 40 Years member

Imrie, Alison Wendy

Member

Jones, David Alun

Fellow

Kenyon, Frank Edwin

Fellow

Lader, Malcolm Harold

Fellow – Over 40 Years member

Lau, Man Pang

Member – Over 40 Years member

Leff, Julian Paul Honorary

Fellow

Lishman, William Alwyn Foundation

Fellow

Maggio, Gabriel I E L

Affiliate

McGovern, Gerald Patrick

Fellow – Over 40 Years member

McLaughlin, Jo-Ann

Member

Mian, Ihsanul Haq

Fellow – Over 40 Years member

Milne, Sheila Johnstone Dedman

Member – Over 40 Years member

Mubbashar, Malik Hussain

Fellow – Over 40 Years member

Nada Raja, Rajaratnam

Fellow – Over 40 Years member

Nadkarni, Pallavi

Member

Palmer, Robert Leslie

Fellow – Over 40 Years member

Pant, Anshuman

Member

Pathak, Rudresh Kumar Dinanath

Member

Pitt, Brice Masterman Norman

Fellow

Price, Michael John

Member – Over 40 Years member

Rana, Mamoona

Pre-Membership Psychiatric Trainee

Roberts, Ann Gertrud

Member

Roberts, David Thomas

Member

Robinson, John Richard

Fellow – Over 40 Years member

Romero Urcelay, Jose Luis

Affiliate

Shah, Ajit Kumar

Fellow

Scorer, Richard Charles

Member – Over 40 Years member

Shafar, Susanne

Fellow

Shirley, Stephanie Honorary

Fellow

Smith, Eileen Dorothy

Fellow – Over 40 Years member

Taylor, David Charles

Fellow – Over 40 Years member

Vallance, Maelor

Fellow – Over 40 Years member

Washbrook, Reginald Alfred Hryhoruk

Fellow – Over 40 Years member

White, Daniel Paul

Member

Wilson, Becky Annabelle

Member

Ashurst, Pamela Margaret

Fellow – Over 40 Years member

Bourne, Stanford

Fellow

El-Hilu, Saleh

Fellow – Over 40 Years member

Freeston, Una

Fellow – Over 40 Years member

Jenkins, Gary Robert

Member

Keating, David Patrick

Member

King, Michael Bruce

Fellow

Simpson, Michael Andrew

Member – Over 40 Years member

Wallace, Daphne Rowena Duckworth

Fellow – Over 40 Years member

Weaving, Hester Claudia

Member – Over 40 Years member

Wells, Peter George

Fellow – Over 40 Years member

Briscoe, Oliver Villiers

Retired

Fellow – Over 40 Years member

Choudry, Nas

Retired

Fellow – Over 40 Years member

Davies, Brian Michael

Retired

Fellow – Over 40 Years member

De-Groot, Michael Henry

Retired Foundation

Member

Fine, Stuart Hamilton

Academic

Fellow – Over 40 Years member

Freed, Edgar David

Consultant

Fellow – Over 40 Years member

Gaitonde, Alison Myra

Retired

Member – Over 40 Years member

Hill, Oscar William

Retired

Fellow – Over 40 Years member

Hore, Brian

Consultant Private Practice

Fellow – Over 40 Years member

Horrocks, Frederick Arthur

Retired

Member

Leff, Julian Paul

Retired Honorary

Fellow

Lishman, William Alwyn Emeritus

Professor Foundation

Fellow

Mian, Ihsanul Haq

Retired

Fellow – Over 40 Years member

Milne, Sheila Johnstone Dedman

Retired

Member – Over 40 Years member

Nada Raja, Rajaratnam

Retired

Fellow – Over 40 Years member

Pitt, Brice Masterman Norman

Professor

Fellow

Romero Urcelay, Jose Luis

Associate Specialist

Affiliate

Shafar, Susanne

Retired

Fellow

